# Physiology

**Published:** 1858-03

**Authors:** 


### Bi-Monthly Periscope.

### PHYSIOLOGY.

1. CIRCULATION OF THE BLOOD AND THE FUNCTIONS OF THE RIGHT AURICLE. By *Thomas Shapter*, *M.D.*, etc.—After a series of careful examinations made upon a man in whom there exists a congenital fissure of the sternum, Dr. Shapter has come to the conclusion "that, as regards the heart, the circulation of the blood is more simply effected than is usually considered to be the case; that, in fact, the heart proper is ventricular, and ventricular only; and that the circulation of the blood through the heart mainly depends upon its ventricular exhaustion and impulsion. The auricle, on the other hand, is rather to be considered as an appendage proper to the venous system, and not as appertaining to the heart; and, without ignoring the influence of the capillaries and of the veins generally, (feeble though this be,) that auricular contraction is not an active agent in the movement of the blood.*

These conclusions I ground on the fact that the whole phenomena witnessed show that the greatest size of the auricle is not induced by a sudden influx of blood from the cavæ,† but on the contraction of the ventricle by the upward pressure of the flaps of the tricuspid valve when propelled into the auriculo-ventricular axis; hence the blood then contained within the auricle is ponded or regurgitated back. The relaxing of the valve and the expansion of the ventricle, whereby the exhausting or suction power of the heart is exercised, immediately relieve the auricle of this undue tension. Its normal condition of a full, but not of an over-distended vessel, is then restored. In this state it continues during its period of rest to receive blood from the cavæ, and to deliver it into the expanding ventricle, till the latter is again filled, and again contracts upon its contents. In the above we see exemplified, as regards the auricle, the phenomena of elasticity rather than those of active muscular contractility, while the ventricle distinctly exemplifies this latter. The normal state of the auricle is that of an open tube containing fluid, and this normal state is disturbed by the hydrostatic force of an increased amount of fluid, but is regained by the elasticity of its coats. Supposing this view to be correct, it is then probable that the musculi pectinati act, firstly, in preventing undue distension, and, secondly, in restoring the auricle to its normal dimensions.

The ordinary explanation of the function of the auricle is that the auricle

---

* This appears to be also the opinion of Dr. Pavy, who says, "The right auricle never empties itself; indeed, with the exception of the appendix, which is but a very small portion of it, it looks like a large sinus, or a bulging between the two cavæ, with no more power of emptying itself than either of these veins."

† In Cruveilhier's case, the large veins near the heart contracted simultaneously with the auricular systole, and not with its diastole, so that they had no influence in causing its dilatation. The same conclusion may be inferred in the case of M. Groux.

contracts on the blood contained within it, and propels it into the ventricle. There are two cogent reasons against this conclusion; first, it would assume that the auricle contains blood enough for the supply of the ventricle, which, as is universally admitted, it does not; and, secondly, the absence of any sufficient valvular apparatus at the portal entrance to the auricle, or where the venæ cavæ deliver themselves into the sinus. This alone would militate against the assumption, for not only is it against the whole principle of the circulation, but in fact the contraction of the auricle would as equally force it into the cavæ as into the ventricle.

Without discussing what may be the motive power exercised upon the blood in its course through the veins, how much may be due to capillary force, and how much to an elastic or even to a contractile power proper to the veins themselves, I here hazard the opinion that the auricle fulfills two evident conditions, the one, in connection with the venæ cavæ and sinus, of being the ready vessel of supply whence the ventricle, or heart proper, receives the blood it requires; the other, that of being an elastic, expanding vessel, and representing one of those enlargements which invariably accompany the valves of the veins, whereby the blow of the ventricle and the sudden contraction of the valves on the incoming stream of blood are compensated for; and that any muscular power it may exert on the propulsion of the blood is only secondary.—*Med. Times and Gazette*, Dec. 26, 1857.

2. ACTION OF THE PANCREATIC JUICE AND BILE IN THE ABSORPTION OF FAT.—Schiff contests, upon pathological grounds, Bernard's view, that the *pancreatic juice* is the only and indispensable agent for the absorption of fat. He adduces some cases in which a considerable amount of fat was discovered in the body of such who had suffered for a long time from disease of the panereas. He further shows that there are cases on record in which the excretion of large quantities of fat-like matter with the fæces had taken place without disease of the pancreas.

Regarding the action of *bile* in the absorption of fat, Schiff contends that the bile does not produce any chemical alteration in the fats, and facilitate, through this, the absorption; but that it acts as a stimulant on the contractile elements of the villi; that it causes them to contract and to empty their contents into the lymphatic vessels; that thus room is prepared for the entrance of a fresh quantity of fat into the emptied villi. The author shows by experiment that bile is a stimulus for the muscular fibre, and especially the organic muscular fibre.—*British and Foreign Medico-Chirurgical Review*, Jan. 1858.

